# Subjective Wellbeing, Objective Wellbeing and Inequality in Australia

**DOI:** 10.1371/journal.pone.0163345

**Published:** 2016-10-03

**Authors:** Mark Western, Wojtek Tomaszewski

**Affiliations:** Institute for Social Science Research and ARC Centre of Excellence for Children and Families over the Life Course, The University of Queensland, Brisbane, Australia; Leibniz Institute for Prvention Research and Epidemiology BIPS, GERMANY

## Abstract

In recent years policy makers and social scientists have devoted considerable attention to wellbeing, a concept that refers to people’s capacity to live healthy, creative and fulfilling lives. Two conceptual approaches dominate wellbeing research. The objective approach examines the objective components of a good life. The subjective approach examines people’s subjective evaluations of their lives. In the objective approach how subjective wellbeing relates to objective wellbeing is not a relevant research question. The subjective approach does investigate how objective wellbeing relates to subjective wellbeing, but has focused primarily on one objective wellbeing indicator, income, rather than the comprehensive indicator set implied by the objective approach. This paper attempts to contribute by examining relationships between a comprehensive set of objective wellbeing measures and subjective wellbeing, and by linking wellbeing research to inequality research by also investigating how subjective and objective wellbeing relate to class, gender, age and ethnicity. We use three waves of a representative state-level household panel study from Queensland, Australia, undertaken from 2008 to 2010, to investigate how objective measures of wellbeing are socially distributed by gender, class, age, and ethnicity. We also examine relationships between objective wellbeing and overall life satisfaction, providing one of the first longitudinal analyses linking objective wellbeing with subjective evaluations. Objective aspects of wellbeing are unequally distributed by gender, age, class and ethnicity and are strongly associated with life satisfaction. Moreover, associations between gender, ethnicity, class and life satisfaction persist after controlling for objective wellbeing, suggesting that mechanisms in addition to objective wellbeing link structural dimensions of inequality to life satisfaction.

## Introduction

Since at least the work of Aristotle, philosophers have been interested in wellbeing, understood as the qualities of a good life or a good society [1]. For Aristotle, living a good life meant achieving one’s potential in knowledge, health, friendship, wealth, and other life domains [1]. Fifteen years ago, Kahneman, Diener and Schwartz [2] proposed a new science of wellbeing focused on explaining positive states of mind and taking seriously people’s expressed subjective assessments of their own emotions and quality of life.

Following Kahneman, Diener and Schwartz’s work [2], social scientists’ and policy makers’ interest in the nature and determinants of wellbeing has grown. Two conceptual approaches to wellbeing research now dominate the field [3–5]. The objective approach defines wellbeing in terms of quality of life indicators such as material resources (e.g. income, food, housing) and social attributes (education, health, political voice, social networks and connections). The subjective approach emphasises subjective wellbeing, that is people’s own evaluations of their lives, especially their life satisfaction (a cognitive evaluation), happiness (a positive emotional state) and unhappiness (a negative emotional state) [1].

The objective approach to wellbeing largely originates from Amartya Sen’s work in welfare economics [6,7]about how to measure poverty and inequality, and its extension [8,9]to the capabilities individuals should have to live fulfilling lives. Political theorists such as Nussbaum [10]have listed core human capabilities to include life, bodily health, bodily integrity, the ability to use the senses to think and to imagine, the ability to express emotions, to exercise practical reason and autonomy with respect to one’s own life, to affiliate, to live with dignity, to live in and with nature, to play, and to control one’s own political and economic environment, through education, work and political and social participation.

The objective approach also informs national and international statistical indicators such as The United Nations Development Programme Human Development Index, the OECD’s Better Life initiative, and the French government’s Commission on the Measurement of Economic Performance and Social Progress [11]. Social indicators frameworks attempt to measure societal development and quality of life using aggregate measures of education, employment, health, housing, income, security, environmental quality and political and social inclusion.

In contrast, the subjective approach conceptualises and measures wellbeing using people’s subjective overall life evaluations [3, 5,12]. Objective and subjective approaches to wellbeing also treat subjective wellbeing (life evaluations) differently. In some objective approaches, subjective wellbeing evaluations are one component of overall wellbeing alongside objective measures and “the core question for the objective approach is to agree on the list of goods that are necessary for a good life” [3]. The subjective approach on the other hand theorises objective wellbeing affecting subjective wellbeing implying that objective goods and circumstances influence subjective life evaluations [3].

These frameworks have had two implications for empirical wellbeing research. First, proponents of the objective approach have not investigated how objective wellbeing indicators influence subjective life evaluations because this question is not theoretically relevant in the objective framework [3]. On the other hand, subjective wellbeing studies have tended to concentrate predominantly on how a small number of objective indicators influence life satisfaction or happiness, with most research examining income [3,4]the predominant objective wellbeing indicator in early research. Some studies examine relationships between subjective wellbeing and other objective indicators such as education [12,13], family and friendship networks [12,14] and health [13]. However, little research examines how subjective wellbeing is related to a comprehensive set of objective wellbeing indicators.

Two recent exceptions are Bohnke and Kohler [3]and Bellani and D’Ambrosio[4]. Bohnke and Kohler [3] use the European Quality of Life Study, a cross-sectional comparative survey of 28 countries, to identify differences in quality of life between European countries and to assess the perceived importance of various dimensions social inequality for people living in different countries. Bellani and D’Ambrosio [4] analyse the European Community Household Panel, a longitudinal survey of EU member states conducted from 1994–2001, to study the relationship between self-reported satisfaction and objective well-being as measured by the indices of deprivation and social exclusion. They find that life satisfaction decreases with an increase in deprivation and exclusion, even when controlling for a range of factors. These papers address particular substantive gaps and have different strengths. Both use high quality survey data and strong measures of objective wellbeing. Including many countries in their analysis enables Bohnke and Kohler [3] to examine the generality of patterns between objective and subjective wellbeing but without longitudinal data they cannot take account of individual changes in objective circumstances or subjective wellbeing, or adjust for unmeasured individual characteristics which might bias their cross-sectional results. Bellani and D’Ambrosio [4] use longitudinal data but cover a more limited set of objective measures than Bohnke and Kohler [3], and lack measures of indicators like health and leisure time. Bellani and D’Ambrosio [4] also lack an overall measure of life satisfaction, and instead use partial indicators measuring satisfaction with work, housing, finances and leisure.

In this paper we attempt to make four research contributions. First, we adopt a broad conception of wellbeing that includes objective dimensions measuring human capabilities in different spheres of life (social, economic, political, environmental), and subjective aspects based in cognitive evaluations of overall life satisfaction. Our objective measures include social, economic and physical components noted by Stiglitz, Sen and Fitoussi [11] and reflect the list of central capabilities proposed by Nussbaum [15] and the comprehensive measures used by Bohnke and Kohler [3]. Second, we use longitudinal rather than cross-sectional data and apply statistical methods that partly adjust for cross-sectional data biases (cf. [16]). Third we also examine how objective and subjective aspects of wellbeing are linked to structural dimensions of inequality, gender, class, age and ethnicity thereby joining the study of wellbeing with the study of social inequality (cf.[3]). Finally, we examine these relationships in Australia, a country that is among the highest in the world in objective wellbeing, but which has not been included in previous comprehensive studies of relationships between subjective and objective wellbeing.

## Research Questions and Hypotheses

We address three distinct but interrelated research questions in this paper:

1. How are objective features of social wellbeing distributed according to socioeconomic and sociodemographic characteristics that indicate categorical relations of inequality or “durable inequalities” of class, gender, age or ethnicity [17]?
2. How are objective aspects of wellbeing related to subjective assessments of social wellbeing (life satisfaction)?
3. How is the relationship between categorical inequalities and subjective wellbeing mediated by objective aspects of wellbeing?

We investigate subjective wellbeing as overall life satisfaction and economic, social and physical objective wellbeing including financial hardship, material deprivation, household income, leisure time, social connections to family and friends, and health. Our structural sources of inequality are class, gender, age and ethnicity, and we formulate specific hypotheses regarding the stratification of objective wellbeing by these factors. Class, gender, age and ethnicity are sources of inequality because social relations associated with them potentially lead to long-lasting systematic differences in social opportunities and rewards [17]. Structural inequalities arise when social processes allocate individuals to social categories with different access to scarce rewards and opportunities [18].

In Australia, as in other societies, gender, class, age and ethnicity systematically shape access to resources, rewards and life-chances. Rewards and outcomes in different social domains are correlated, and advantages and disadvantages therefore potentially compound over the lifecourse [19]. For instance, socioeconomic and class differences in the family of origin are associated with socioeconomic differences in educational achievement[20], and health [21] which themselves are associated with variations in employment outcomes in later life [22]. We expect those privileged by gender, class, age and ethnicity to be generally advantaged with respect to objective measures of wellbeing. However we also anticipate particular links between some inequality dimensions and some objective aspects of wellbeing.

Class relations are fundamentally linked to the economic conditions of people’s lives, whether through social closure linked to market based skills and educational qualifications, or relationships of domination and exploitation associated with owning and controlling economic resources such as property, economic capital, or organisational resources [23,24]. We expect class relations to be most strongly related to economic aspects of objective wellbeing with more privileged classes being more advantaged economically than less privileged classes (Hypothesis 1a).

In contemporary societies gender relations stratify economic [25], and social outcomes [26] to favour men over women. Gender inequality reflects various mechanisms including opportunity hoarding by men, cultural norms about gender-specific appropriate behaviours (e.g.[27]) and beliefs about male and female competence and capability [18,26]. While countries differ in the degree of gender inequality in objective wellbeing, comprehensive analyses such as the World Economic Forum’s Global gender gap index [28] find gender differences in objective wellbeing indicators in all countries studied. We therefore expect men to be advantaged over women with respect to most objective aspects of wellbeing (Hypothesis 1b).

We expect age stratification with respect to objective wellbeing for reasons noted by life course theorists: life changes take place over long periods of human lives with prior life history affecting later life outcomes; life course processes occur across multiple domains of life such as work and family [29]. These arguments imply that life course structured outcomes occur throughout people’s lives across multiple domains and that outcomes in different domains are linked because prior events and experiences shape subsequent outcomes. The life course approach does not translate into simple predictions about relationships between age and objective wellbeing, but does imply we will find age-based distributional inequalities in objective wellbeing (Hypothesis 1c). For some wellbeing indicators research suggests particular patterns, such as the curvilinear profile of earnings with age, found in economic research or older people’s greater leisure time [30]. For other indicators, such as financial hardship or material deprivation, empirical predictions are not clear-cut.

Ethnicity is a categorical source of inequality in Australia, notably through the disadvantage experienced by Indigenous Australians, but also through its links to English language proficiency among migrants. Indigenous status is a profound source of inequality on “almost any conceivable measure of socio-economic wellbeing” [31], in part because Indigenous inequality is a “wicked problem” [32], in which policy and program delivery is inherently difficult [33]. Australia monitors Indigenous inequality in objective wellbeing with “Closing the Gap” targets for life expectancy, child mortality, education and employment. Indigenous-non-Indigenous gaps exist on all indicators and on some such as life expectancy, literacy, numeracy, and employment, there has been no progress to address inequality since monitoring started [34]. We expect Indigenous status to be associated with negative wellbeing on all objective measures (Hypothesis 1d).

Second, English is the national language of Australia and English proficiency is linked to both economic and social outcomes [35]. Differences in English proficiency for instance, explain a large part of the wage gap between immigrants and the native born [36]. We expect non-English speaking background to be negatively related to all objective wellbeing dimensions (Hypothesis 1e).

We also investigate how categorical inequalities and objective aspects of social wellbeing are related to life satisfaction. Conceptually, life satisfaction is an overall subjective assessment of wellbeing arising from the different circumstances and conditions of one’s life. Material wellbeing, work, health, leisure, social and family connections all influence subjective wellbeing [37] implying that objective wellbeing is positively associated with subjective wellbeing. Previous research finds that income and subjective wellbeing are related with richer people more satisfied with their lives than poorer people [4,38]. In their comprehensive analysis, Bohnke and Kohler [3] also find other elements of objective wellbeing such as contact with friends and neighbors, health, and housing are positively associated with life satisfaction.

Research also suggests that some categorical inequalities, notably gender, age and class, are associated with subjective wellbeing. In particular, middle aged people consistently report less satisfaction with their lives than younger or older people [3,13,39], while work characteristics such as pay, job security, autonomy, and authority have been linked to higher job satisfaction, and thus higher overall life satisfaction [3]. The latter results suggest that individuals in more privileged classes will report higher overall life satisfaction than those in more disadvantaged classes. Women also report higher levels of life satisfaction than men [3,40]. We therefore hypothesise that men, individuals in less privileged classes, and those in their middle ages will report lower satisfaction with life, compared with other groups (Hypothesis 2a).

We are not aware of any studies comparing life satisfaction of Indigenous and non-Indigenous Australians, although to the extent that subjective wellbeing is linked to objective circumstances, we hypothesise that Indigenous Australians will be less satisfied with their lives than non-Indigenous Australians, at least before controlling for objective wellbeing (Hypothesis 2b). For a similar reason we hypothesise that migrants from a non-English speaking background will be less satisfied with their lives than the Australian born and migrants from English speaking countries (Hypothesis 2c).

By incorporating direct measures of objective well-being in different domains, along with categorical measures of inequality, we are able to examine whether or not categorical inequalities are associated with subjective wellbeing directly or indirectly, through their effects on objective dimensions of wellbeing. Given that life satisfaction is an overall cognitive evaluation reflecting the circumstances of one’s life, we hypothesise that there will be strong direct relationships between objective measures of wellbeing and life satisfaction (Hypothesis 3a). However, independently of objective circumstances, we do not expect categorical inequalities to directly influence life satisfaction beyond and above the association through objective wellbeing (Hypothesis 3b).

Our empirical analysis relies on 3 waves of a longitudinal household panel study conducted in Queensland, the third largest state of Australia. Australia is a pertinent case in which to investigate inequality in objective and subjective wellbeing. For the last five years, Australia has ranked second behind Norway on the Human Development Index, a composite measure of life expectancy, income and education, and since 1980 Australia has typically ranked either second or first [41]. Moreover Australia also ranks second on the Inequality Adjusted HDI [41], which incorporates inequalities in each of the three dimensions of wellbeing (education, life expectancy, income) measured by the HDI. Previous studies of cross-national differences in the relationship of income to subjective wellbeing have found that within countries income is positively associated with life satisfaction, but also that those in richer countries are more satisfied than those in poorer countries [4]. If the income findings generalise to other elements of objective wellbeing, we might expect to Australia to show similar within country patterns in objective and subjective wellbeing as other countries, while also showing a high level of overall subjective wellbeing. In other words, if we find robust associations between objective and subjective wellbeing, we might expect these associations also to hold in other countries.

## Data and Methods

### Data and sample

We use data from the Living in Queensland Social Wellbeing Study, a longitudinal Australian panel survey that started in 2008 and followed a representative sample of Queensland households. The study is designed to operationalise and examine multidimensional inequality and wellbeing. The sample covers respondents aged 18 and over living in private households (including multiple-household dwellings). At the first wave of the survey, one person per household was selected using random sampling stratified by region, age and gender and this person completed the Personal form. A person from the sampled household was then asked to provide information about the household as a whole. The respondents to the personal questionnaire were followed over the course of three annual interviews (2008, 2009, and 2010) with complementary household information obtained at each wave. In total, 3,959 respondents provided answers to the survey in wave 1, 2,723 in wave 2, and 2,360 in wave 3, including those who partially completed it.

The study participants were recruited through a brief telephone interview. Trained interviewers conducted a five to ten minute telephone interview to make initial contact with householders, provide information on what the study involves and recruit voluntary study participants (randomly selected from the household members). Verbal consent was obtained for participation in the study and willing participants provided their name and contact details to enable the survey forms to be sent out to the household. Verbal, rather than written, consent was sought at the recruitment stage, as the process used landline phone numbers as a sampling frame. The consent was recorded in the Computer Assisted Telephone Interview (CATI) system. The procedure followed the National Health and Medical Research Council’s ethical guidelines and the privacy legislation. Once consent to participate had been obtained, the participants were sent an information sheet and survey pack (a paper or online questionnaire). Furthermore, the following text was included in the information sheet and survey forms to confirm participant’s consent “Completing and returning the survey to us will be taken as your consent to participate in this study” and the participants were informed that they could freely withdraw from the project at any time. The process of obtaining consent and recruiting the study participants was approved by the Behavioural & Social Sciences Ethical Review Committee of the University of Queensland.

The survey is representative of all households in the Australian state of Queensland. Queensland is the third largest state in Australia, containing approximately 20 per cent of the country’s population. It includes the fastest growing population region in the country, largely because of internal migration linked to the strong state economy. In terms of age and sex, Queensland is highly representative of Australia. In 2009, the middle year of data collection, the Queensland median age was 36.2 years, while the median age of the Australian population was 36.9 years [42]. Queensland’s sex ratio in 2009 was 100.0 while the Australian sex ratio was 99.2 [42]. Compared with other Australian states and territories, Queensland sits in the middle in terms of both mean income and levels of inequality. For example, the equivalised disposable household income (person weighted) in 2007/2008 was $810 per week in Queensland, while it was $811 per week in all of Australia [43].

We used an unbalanced panel design to construct our analytic sample, removing partially completed surveys where no information was provided on the items used to construct the dependent variables in our analyses. This resulted in 7,987 person-year observations used for analyses in this paper: 3,367 persons interviewed in wave 1 (85% of the full wave 1 sample), 2,403 in wave 2 (88% of the full wave 2 sample), and 2,217 in wave 3 of the survey (94% of the full wave 3 sample). The analytic sample has a socio-demographic profile broadly reflecting the Queensland population: the median age in this adult-only sample is 52; the median annual household income is about $70,000; however, women are somewhat over-represented in the survey (58%). Even though the respondents were initially approached by telephone, the size of population without a working landline telephone in Australia at the time of the survey was generally considered to be small enough not to cause significant bias to population estimates. The data from the Australian Communications Media Authority show that 94% Australian households had a landline telephone connection in 2008 [44]. The results presented in the paper are based on unweighted data, however, the statistical models include a number of variables typically associated with non-response and attrition.

### Key variables

Table 1 describes the key measures used in the paper—the indicators of objective and subjective wellbeing. Our subjective wellbeing indicator is the *Satisfaction With Life Scale* (SWLS) [45], a widely used and well-validated instrument (e.g. [46]). The scale has a highly satisfactory internal consistency in our data, as indicated by Cronbach’s alpha of 0.91.

**Table 1:** Wellbeing indicators.

Dimension of wellbeing	Measure
***Subjective wellbeing***	
*Life satisfaction*	Average of five items measured on a 7-point scale each (Satisfaction With Life Scale (SWLS)): (*In most ways my life is close to my ideal; The conditions of my life are excellent; I am Satisfied with life in general; So far I have gotten the important things I want in life; If I could live my life over*, *I would change almost nothing*) (higher score = more satisfied)
***Objective wellbeing***	
*Income*	Log total household income (self-reported): before tax, last financial year, equivalised using square root household size;
*Financial hardship*	Count of the number of problems over the past 12 months from a list of 5 items (*Couldn’t keep up with payments for water*, *electricity*, *gas or telephone; Got behind with the rent or mortgage; Moved house because the rent/mortgage was too high; Had to pawn or sell something*, *or borrow money from a money lender; Had to ask a welfare agency for food*, *clothes accommodation or money*). (higher score = more hardship)
*Material deprivation*	Average score based on eight items measuring frequency the respondents’ family could not afford the following goods or services over the past 12 months on a 4-point scale (*Warm clothes and bedding if it is cold; Decent meal; Medicines; A decent and secure home; Heating in at least one room of the house; Outings with friends; Visits to a doctor when you or a family member was sick; Visits to a dentist when you or a family member needed to*). (higher score = more deprived)
*Leisure time*	Log leisure time (in hours per week)
*Health*	Self-reported health status, measured on a 5-point scale (*Excellent*, *Very good*, *Good*, *Fair*, *Poor*). (higher score = better health)
*Contacts with family*	Self-reported variable measuring how often the respondent spends time with parents children or other relatives, measured on a 6-point scale (higher score = more contact)
*Contacts with friends*	Self-reported variable measuring how often the respondent spends time with their friends measured on a 6-point scale (higher score = more contact)
***Indicators of events potentially affecting wellbeing***	
*Negative events*	Number of the following events experienced over the past 12 months: *Family illness; Lost job; Experienced a major financial crisis; Failed an important exam; Serious illness; Separated; Immediate family member died; Close family member died; A friend died; Was a victim of a property crime; Was assaulted; Served a prison sentence; Family member served a prison sentence*.
*Positive events*	Number of the following events experienced over the past 12 months: *Was promoted; Got married; Passed an important exam; Reconciled with a partner; Gave birth/adopted a child (either respondent or the partner*.

Indicators presented in Table 1 are valid measures of objective wellbeing, despite being reported by the respondent, rather than obtained from independent sources. Each one provides a description of respondent’s status, rather than an evaluation of how happy or satisfied the respondent is with this status, which would be measuring subjective wellbeing. Even self-reported health is widely used as a health status measure of psychosocial and biological health in epidemiological and social surveys [47,48]. The objective aspects of wellbeing are included as individual indicators in our models rather than combined into an aggregate index as in some previous work (e.g. [4]). We are interested in how components of objective wellbeing are distributed among different groups, and also potentially relate differently to subjective wellbeing. Correlation analysis confirms that the dimensions of objective wellbeing are largely independent of each other, displaying very moderate associations, with almost all correlation coefficients lower than +/- 0.25 and a vast majority not significant at 0.05 level; the only exception is somewhat higher correlation between material deprivation and reported financial hardship (+0.44).

We also included two key control variables in regression models predicting subjective wellbeing: indexes of positive and negative events that the respondents experienced over the past 12 months. In Western societies, responses to general subjective wellbeing or life satisfaction questions tend reflect “homeostasis”; most people report positive life satisfaction with a tendency to return to the same values over time (a “set point”). The homeostatic “set point” for individuals can be altered in the short term by happy or sad events [49], and in the long term [50]by major life events such as repeated unemployment [51]or marriage [52]. To address this issue we control for a number of positive and negative life events.

Our analysis of categorical inequalities focuses particularly on gender, class, age and ethnicity, as captured by non-English Speaking background and Indigenous status, with additional socio- demographic control variables that are likely related to objective and subjective wellbeing. The key predictors of wellbeing in our analyses are:

*Gender* (Male, Female);

*Age* (coded as a categorical variable: 17–34, 35–44, 45–54, 55–64, 65+);

*Class*–based on labour force status, employment relations if employed, and occupation and skill level as classified by the Australian and New Zealand Standard Classification of Occupations [53]. The measure has connections to the employment relations based account of Goldthorpe [23] and the relations of production account of Wright [54]. The class categories are Employers (self-employed and having employees), Petty bourgeoisie (self-employed & working on their own), Skilled managers (ANZSCO Major Group 1; skill Level 1), Other managers (Major Group 1; other skill levels), Professionals (Major Group 2), Skilled technical (Major Group 3; skill Level 1), Other technical (Major Group 3; other skill levels), Skilled white collar (Major Groups 4,5 & 6; skill Level 2), Other white collar (Major Groups 4,5 & 6; other skill levels), Skilled blue collar (Major Groups 7 & 8; skill Level 4), Other blue collar (Major Groups 7 & 8; other skill levels), Not working

*Aboriginal or Torres Strait Islander* Status (ATSI; Yes, No)

*Non-English Speaking Background* (NESB; Yes, No); This variable proxies English language proficiency, which is not measured directly in our data, but also captures more broadly cultural diversity. About 30 per cent of Wave 1 respondents of non-English speaking background were born in Australia and many of those who were born overseas had been living in the country for many years (median was 28 years).

Our control variables include marital status, presence of children in the household (dependent children under 18; and preschool children under 6), education, labour force attachment (whether work is main activity of the respondent) and home ownership status.

We initially screened for outliers and inconsistencies. We then used Confirmatory Factor Analysis on pooled sample (performed in Mplus) to test whether the measures of financial difficulties and material deprivation each formed a single underlying construct. The results were satisfactory, with both measures achieving high values of goodness-of-fit statistic (financial hardship: RMSEA = 0.02, CFI>0.99; TLI = 0.99; material deprivation: RMSEA = 0.05, CFI = 0.99; TLI = 0.99) and acceptable reliability (financial hardship: alpha = 0.65; material deprivation: alpha = 0.81).

To minimise the loss of data, we imputed missing values on the measures of wellbeing. The proportion of imputed values was in most cases under 5 per cent; the exceptions included material deprivation (7.4 per cent), income (10.6 per cent) and leisure time (21.0 per cent). We used two methods of imputation: mean-values and within-person averages based on the data available for the same individual in other waves. Our statistical models included dummy variables indicating imputation as control variables. We do not report coefficients for imputation controls in the regression tables because, with one exception, their effects were not statistically significant; we note the exception in the relevant place.

### Empirical strategy

We begin by inspecting distributional features of objective and subjective wellbeing. Next we use regression models to examine relationships between the sociodemographic variables and the wellbeing measures to provide information about sociodemographic distribution of social wellbeing. Gender, class, age and ethnic variations in objective wellbeing provide evidence of durable categorical inequalities. Finally, we regress subjective wellbeing on objective wellbeing and sociodemographic variables, including our measures of categorical inequalities. These last analyses enable us to assess whether objective differences in wellbeing are mechanisms that link sociodemographic inequalities to differences in life satisfaction.

The main analytical method used in the paper is a mixed effects hybrid model for longitudinal data [55], which can be expressed as:

$$Y_{ij}=\alpha_{j}+\beta_{1}(X_{ij}-{\bar{X}}_{i})+\beta_{2}{\bar{X}}_{i}+\;\beta_{3}Z_{i}+\;\mu_{i}+v_{ij}$$

where X_ij_ and Z_i_ represent sets of time-varying and time-invariant predictors.

In a longitudinal dataset for different individuals observed at different times (survey waves), there are two sources of variation in the response variable. The between-individual variation is the variation in respondents’ mean values (i.e. averaged over time) on the dependent variable. The within-individual variation is the variation that a single respondent’s time-specific score exhibits around his/her mean response score. A standard random effects estimator produces regression coefficients that are a weighted average of the between-individual and within-individual variation. A hybrid model extends a random effects model by transforming the original independent variables into group-mean deviations and adding their group-means as additional independent variables. This provides a way of relaxing the assumption in the random-effects estimator that observed variables are uncorrelated with the unobserved variables, which was originally proposed by Mundlak [56]and allows estimates of the between and within-effects. A routine to estimate hybrid models is available in Stata [57].

In the results section, we decompose the total variance into the between and within components to gain insights into the cross-sectional and temporal distribution of inequalities in wellbeing, and subsequently present the between- and within- effects estimated by the hybrid models. Our analytic strategy combines random and fixed effects modelling frameworks, controlling for unobserved, time-invariant characteristics of individuals, such as psychological profiles, depression, anxiety or self-esteem and yielding coefficients for some indicators of durable categorical inequalities, such as gender or ethnicity, which are stable over time and therefore not estimated by the fixed effects model. However, being based on observational data, it is important to note that while the estimated effects reflect statistical associations, they do not necessarily imply causality.

## Results

### Investigating the socio-demographic distribution of objective wellbeing

Table 2 shows two indices of inequality—the relative mean deviation and the coefficient of variation—calculated for all wellbeing dimensions. The relative mean deviation represents an average of individual deviations of wellbeing scores from the mean for the whole population, while the coefficient of variation is the ratio of the standard deviation to the mean for each wellbeing score. For a detailed discussion of these and other measures of inequality, see for instance [58]. Financial hardship is most unequally distributed largely because it is a count variable. The dimension of objective wellbeing that is most subject to direct policy interventions, income, shows least inequality. However, when material situation is measured using a material deprivation index, we observe markedly higher inequality. Inequality in life satisfaction is also high—on par with inequalities the distribution of health status or the frequency of contacts with family.

**Table 2:** Selected inequality measures of objective and subjective wellbeing.

	Relative mean deviation	Coefficient of variation
*Income*	0.07	0.20
*Material deprivation*	0.12	0.37
*Financial hardship*	0.79	2.68
*Health*	0.12	0.30
*Leisure time*	0.09	0.27
*Contacts w/ family*	0.14	0.33
*Contacts w/ friends*	0.10	0.28
*Life satisfaction*	0.13	0.32

The second stage of the analysis involved investigating how the objective wellbeing dimensions are distributed according to key socio-demographic characteristics. We first decomposed total variance for each outcome variable into between-person and within-person components to gain insights into cross-sectional and temporal variation in objective wellbeing in our data (Table 3).

**Table 3:** Decomposition of variance for objective wellbeing indicators.

	Income	Financial hardship	Material depriv	Health	Leisure	C w/ family	C w/ friends
**Variance**							
*Between-persons*	*0*.*65*	*0*.*51*	*0*.*35*	*0*.*83*	*0*.*55*	*1*.*24*	*0*.*84*
*Within-persons*	*0*.*41*	*0*.*35*	*0*.*29*	*0*.*55*	*0*.*65*	*0*.*88*	*0*.*73*
*ICC*	*0*.*71*	*0*.*68*	*0*.*58*	*0*.*70*	*0*.*41*	*0*.*66*	*0*.*57*

There is more variation in objective wellbeing between persons than variation over time for the same persons, as shown by intra-class coefficient (ICC), all of which, with the exception of leisure time, have values over 0.5. This is not surprising as the observation period in our study is relatively short (3 years). Most objective components of wellbeing are rather stable over this time period. Despite this, there is still considerable within-person variation on all indicators, as measured by (1-ICC), ranging from about 30% for health to 59% for leisure time. Objective wellbeing is therefore quite fluid showing some variability over time in all dimensions.

We next estimated a hybrid model for each of the wellbeing measures. The models we estimate assume that the dependent variable is linear, which is potentially problematic for some of our measures of objective wellbeing, particularly financial hardship, material deprivation, health, contact with family, and contact with friends. To check the robustness of the estimates against non-linearity, we re-estimated models for these variables using ordinal random effects logit procedure and compared them with corresponding random effects linear models. The results of these analyses were substantively the same: the direction of all associations remained the same and all coefficients that were statistically significant in the linear model remained statistically significant in the ordinal logit model. This is consistent with findings reported previously in the wellbeing literature, whereby the error introduced by assuming cardinality for ordered variables has been shown to be negligible [59]. Table 4 presents the ‘between’ and ‘within’ effects estimated by the hybrid model for our key indicators of categorical inequalities: gender, age, class and ethnicity. We report unstandardized regression coefficients.

**Table 4:** Between and within effects from mixed-effects hybrid regression models on objective wellbeing indicators.

	Income	Financial hardship	Material depriv	Health	Leisure	C w/ family	C w/ friends
***BETWEEN EFFECTS***							
**Female**	-0.05*	0.03	0.05**	0.08*	-0.17***	0.14**	-0.05
	(0.02)	(0.02)	(0.01)	(0.03)	(0.03)	(0.04)	(0.04)
**Age**							
*17–34*	ref	ref	ref	ref	ref	ref	ref
*35–44*	-0.05	-0.07	-0.07	-0.03	0.03	0.11	-0.11
	(0.06)	(0.05)	(0.04)	(0.08)	(0.10)	(0.13)	(0.11)
*45–54*	-0.00	-0.13*	-0.07	-0.16	-0.03	0.05	-0.27
	(0.08)	(0.07)	(0.05)	(0.10)	(0.12)	(0.17)	(0.14)
*55–64*	0.02	-0.12	-0.13*	-0.25*	0.02	-0.10	-0.37*
	(0.09)	(0.08)	(0.06)	(0.12)	(0.15)	(0.19)	(0.16)
*65+*	-0.02	-0.10	-0.16*	-0.33*	0.13	-0.19	-0.28
	(0.10)	(0.09)	(0.07)	(0.14)	(0.17)	(0.23)	(0.19)
**Class**							
*Petty bourgeoisie*	0.10	-0.07	0.03	0.10	-0.00	-0.14	-0.23*
	(0.06)	(0.05)	(0.04)	(0.08)	(0.10)	(0.13)	(0.11)
*Employer*	0.24***	-0.04	0.01	-0.02	-0.17*	-0.29*	-0.16
	(0.05)	(0.05)	(0.04)	(0.07)	(0.09)	(0.11)	(0.09)
*Skilled manag*.	0.20***	-0.03	-0.01	0.01	-0.25**	-0.42***	-0.18
	(0.06)	(0.05)	(0.04)	(0.08)	(0.09)	(0.12)	(0.10)
*Other managers*	0.28**	-0.04	-0.06	0.13	-0.02	0.01	-0.07
	(0.09)	(0.07)	(0.06)	(0.12)	(0.14)	(0.19)	(0.15)
*Professionals*	0.17***	0.03	0.01	0.09	-0.12	-0.15	-0.17*
	(0.05)	(0.04)	(0.03)	(0.07)	(0.08)	(0.10)	(0.09)
*Skilled tech*.	0.16	0.03	0.04	-0.16	-0.16	-0.06	-0.18
	(0.09)	(0.07)	(0.06)	(0.11)	(0.14)	(0.18)	(0.15)
*Other technical*	0.21**	0.05	0.04	0.20	-0.27*	-0.36*	-0.39**
	(0.08)	(0.06)	(0.05)	(0.10)	(0.12)	(0.16)	(0.13)
*Skilled white-c*	0.20**	-0.02	-0.01	0.06	-0.15	-0.27*	-0.19
	(0.07)	(0.06)	(0.05)	(0.09)	(0.10)	(0.14)	(0.12)
*Other white-c*	0.14**	0.08*	0.08**	0.03	-0.22**	-0.17	-0.26**
	(0.04)	(0.04)	(0.03)	(0.06)	(0.07)	(0.10)	(0.08)
*Skilled blue-c*	0.16*	-0.03	-0.00	0.28**	0.07	-0.07	-0.11
	(0.08)	(0.07)	(0.06)	(0.11)	(0.13)	(0.17)	(0.14)
*Other blue-c*	0.24***	-0.10	-0.01	0.02	-0.13	-0.24	-0.25*
	(0.07)	(0.06)	(0.05)	(0.09)	(0.10)	(0.14)	(0.12)
*Not working*	ref	ref	ref	ref	ref	ref	ref
**ATSI**	-0.23*	0.32***	0.10	-0.21	-0.15	-0.26	-0.06
	(0.09)	(0.09)	(0.06)	(0.15)	(0.11)	(0.19)	(0.16)
**NESB**	-0.16***	0.02	0.13***	-0.00	-0.20***	0.13	-0.11
	(0.04)	(0.04)	(0.03)	(0.07)	(0.05)	(0.08)	(0.07)
***WITHIN EFFECTS***							
**Female**	.	.	.	.	.	.	.
**Age**							
*17–34*	ref	ref	ref	ref	ref	ref	ref
*35–44*	-0.04	-0.06	0.04	-0.22***	-0.01	-0.11	-0.15*
	(0.04)	(0.03)	(0.03)	(0.06)	(0.04)	(0.08)	(0.06)
*45–54*	-0.02	-0.04	0.04	-0.31***	0.07	-0.07	-0.24***
	(0.04)	(0.04)	(0.03)	(0.06)	(0.05)	(0.08)	(0.07)
*55–64*	-0.10*	-0.13***	-0.02	-0.26***	0.15**	-0.44***	-0.27***
	(0.04)	(0.04)	(0.03)	(0.07)	(0.05)	(0.09)	(0.07)
*65+*	-0.27***	-0.19***	-0.07*	-0.25***	0.31***	-0.67***	-0.16
	(0.05)	(0.04)	(0.03)	(0.08)	(0.06)	(0.10)	(0.08)
**Class**							
*Petty bourgeoisie*	-0.01	0.09	0.07	0.22	-0.26**	-0.01	-0.01
	(0.08)	(0.07)	(0.05)	(0.12)	(0.09)	(0.16)	(0.14)
*Employer*	0.28***	-0.03	-0.04	0.41***	-0.23**	0.11	-0.00
	(0.07)	(0.06)	(0.05)	(0.11)	(0.08)	(0.14)	(0.12)
*Skilled manag*.	0.48***	-0.10	-0.08	0.30**	-0.13	0.09	-0.10
	(0.07)	(0.07)	(0.05)	(0.11)	(0.09)	(0.15)	(0.13)
*Other managers*	0.21	-0.03	-0.03	0.24	-0.06	0.18	0.30
	(0.11)	(0.10)	(0.07)	(0.17)	(0.13)	(0.22)	(0.19)
*Professionals*	0.27***	-0.11*	-0.01	0.31***	-0.12	0.12	-0.11
	(0.06)	(0.06)	(0.04)	(0.09)	(0.07)	(0.12)	(0.10)
*Skilled tech*.	0.23*	-0.19*	-0.04	0.11	0.05	0.59**	0.07
	(0.10)	(0.10)	(0.07)	(0.16)	(0.12)	(0.21)	(0.18)
*Other technical*	0.11	-0.08	-0.06	0.27*	-0.07	-0.05	-0.10
	(0.07)	(0.07)	(0.05)	(0.12)	(0.09)	(0.15)	(0.13)
*Skilled white-c*	0.34***	-0.12	-0.00	0.20	-0.06	-0.08	-0.13
	(0.08)	(0.07)	(0.05)	(0.12)	(0.09)	(0.15)	(0.13)
*Other white-c*	0.14*	-0.05	-0.00	0.28**	-0.03	0.15	-0.06
	(0.06)	(0.05)	(0.04)	(0.09)	(0.07)	(0.11)	(0.10)
*Skilled blue-c*	0.11	0.05	0.08	0.17	-0.11	-0.16	-0.10
	(0.08)	(0.07)	(0.05)	(0.12)	(0.09)	(0.16)	(0.14)
*Other blue-c*	-0.10	0.07	0.07	0.28*	-0.02	-0.14	-0.12
	(0.08)	(0.07)	(0.05)	(0.12)	(0.09)	(0.16)	(0.13)
*Not working*	ref	ref	ref	ref	ref	ref	ref
**ATSI**	.	.	.	.	.	.	.
**NESB**	.	.	.	.	.	.	.
Constant	3.84***	0.10	1.06***	3.41***	2.95***	4.22***	4.18***
*N*	7987	7987	7987	7987	7987	7987	7987

Note: The models also includes controls for marital status; the presence of children in the households; education; labour market attachment and tenure.

As expected, there is a strong class gradient to income (Hypothesis 1a). The within and between estimators suggest that skilled managers and professionals, and skilled white-collar workers have higher average incomes and better health. However, they also have less leisure time and less frequent contacts with family than other groups. Moving between classes is also associated with changes in financial hardship. In particular, post-hoc tests confirm that moving between blue collar classes and small-scale self-employment, on one hand, and professional and skilled technical classes on the other is associated with less financial hardship.

Also as hypothesised (Hypothesis 1b), many dimensions of objective wellbeing are stratified by gender: women are disadvantaged in terms of income, report higher levels of material deprivation and spend less time on leisure. They do, however enjoy better health than men, and have more frequent contacts with family.

The within-person estimator suggests significant changes in individuals’ circumstances as they move through the lifecourse (the between-person estimator shows consistent albeit weaker associations) as predicted by Hypothesis 1c. Reported health status and contacts with friends worsen with age, compared to the youngest age group. The oldest age groups, particularly those over 65, also experience a drop in their incomes as well as less frequent contacts with family. Older people, however, have more leisure time than younger age groups, supporting previous US research [30]. Older people also report fewer financial problems and less material deprivation, a pattern found previously in research on poverty (e.g.[60]), and typically explained by changes in expectations for, and perceptions of, their standard of living, or the effects of unmeasured variables such as other wealth or savings.

Ethnic inequality is also present, with Indigenous respondents having lower equivalised income than non-Indigenous respondents and more financial hardship (consistent with Hypothesis 1d). People of non-English speaking background report higher material deprivation, lower income and less leisure time than English speakers (in line with Hypothesis 1e).

### Analysing the associations between objective and subjective wellbeing

In the final analysis stage we investigate the link between objective and subjective wellbeing. We also explore how the relationship between durable categorical inequalities and subjective wellbeing is mediated by objective aspects of wellbeing. Table 5 presents two hybrid models: Model 1 only contained baseline socio-demographic characteristics of respondents as predictors of subjective wellbeing, while Model 2 included objective wellbeing indicators. Again, we report unstandardized coefficients, which can be interpreted as raw differences in the life satisfaction (measured on a 7-point scale) due to the differences in the values of independent variables (as compared to their respective reference categories).

**Table 5:** Between and within effects from mixed-effects hybrid regression models on subjective wellbeing (Satisfaction with Life Scale).

	*BETWEEN EFFECTS*		*WITHIN EFFECTS*	
	Model 1	Model 2	Model 1	Model 2
**Female**	0.13**	0.16***	.	.
	(0.05)	(0.04)		
**Age**				
*17–34*	ref	ref	ref	ref
*35–44*	0.00	-0.01	-0.28***	-0.12
	(0.14)	(0.14)	(0.08)	(0.07)
*45–54*	0.01	0.04	-0.43***	-0.22**
	(0.17)	(0.17)	(0.08)	(0.08)
*55–64*	-0.06	-0.02	-0.23*	-0.05
	(0.21)	(0.20)	(0.09)	(0.09)
*65+*	-0.14	-0.10	0.06	0.14
	(0.24)	(0.24)	(0.10)	(0.10)
**Class**				
*Petty bourgeoisie*	-0.21	-0.22	0.52**	0.52***
	(0.14)	(0.14)	(0.17)	(0.15)
*Employer*	-0.04	-0.02	0.25	0.08
	(0.12)	(0.12)	(0.15)	(0.13)
*Skilled managers*	-0.03	-0.02	0.21	0.02
	(0.13)	(0.13)	(0.16)	(0.14)
*Other managers*	-0.01	-0.04	-0.25	-0.44*
	(0.20)	(0.20)	(0.23)	(0.21)
*Professionals*	-0.10	-0.10	0.30*	0.15
	(0.11)	(0.11)	(0.13)	(0.12)
*Skilled technical*	-0.03	0.02	0.09	-0.08
	(0.19)	(0.19)	(0.22)	(0.20)
*Other technical*	-0.34*	-0.33	0.19	0.06
	(0.17)	(0.17)	(0.16)	(0.15)
*Skilled white-c*	-0.13	-0.13	0.25	0.15
	(0.15)	(0.15)	(0.16)	(0.15)
*Other white-c*	-0.08	-0.05	0.20	0.07
	(0.10)	(0.10)	(0.12)	(0.11)
*Skilled blue-c*	0.16	0.10	0.09	0.10
	(0.18)	(0.18)	(0.17)	(0.15)
*Other blue-c*	-0.12	-0.10	0.10	0.07
	(0.15)	(0.15)	(0.17)	(0.15)
*Not working*	ref	ref	ref	ref
**ATSI**	0.26	0.51**	.	.
	(0.20)	(0.18)		
**NESB**	-0.10	-0.02	.	.
	(0.09)	(0.08)		
**Income**		0.03		0.05
		(0.03)		(0.03)
**Financial hardship**		-0.07		-0.08*
		(0.04)		(0.04)
**Material deprivation**		-0.15**		-0.47***
		(0.05)		(0.06)
**Health**		0.16***		0.38***
		(0.03)		(0.02)
**Leisure**		0.05*		0.13***
		(0.02)		(0.03)
**Contacts w/ family**		0.02		0.03*
		(0.02)		(0.02)
**Contacts w/ friends**		0.06**		0.17***
		(0.02)		(0.02)
**Positive events**		-0.02		0.11*
		(0.03)		(0.05)
**Negative events**		0.01		-0.08***
		(0.02)		(0.02)
Constant	4.91***	3.18***	4.91***	3.18***
*N*	7987	7987	7987	7987

Note: The models also includes controls for marital status; the presence of children in the households; education; labour market attachment and tenure

Model 1 reveals associations between higher subjective wellbeing and categorical inequalities that are consistent with previous research. The within estimator predicts a u-shaped relationship between age and life satisfaction, corroborating the pattern typically reported by others [39] and consistent with our Hypothesis 2a. All else being equal, women report higher subjective wellbeing, despite being objectively disadvantaged (cf. Table 4), which mirrors earlier Australian findings [49]. There are also within effects for class with movements between professional and petty bourgeois classes and not working being associated with higher life satisfaction, and movements between professional and petty bourgeois locations and other managerial classes associated with declining life satisfaction. These findings are also consistent with our expectations, as outlined in Hypothesis 2a.

The within effects for Model 2 reveals strong positive associations between several objective measures of wellbeing and life satisfaction, supporting our Hypothesis 3a. Better health, more leisure time, more frequent contacts with family and friends, and less material deprivation and financial hardship were all associated with higher subjective wellbeing. Consistent with our expectations, we also find a statistically significant association between positive events and increased life satisfaction, and between negative events and lower life satisfaction.

However, income did not have an independent effect on life satisfaction, after accounting for other aspects of objective wellbeing. The absence of an income effect is noteworthy, because the cross-sectional relationship between individual income and subjective wellbeing is a strong empirical finding [4] despite debate about whether income and life satisfaction are related [61]. We do not know of other longitudinal studies that also control for changes in deprivation and financial hardship when they measure shifts in relative income. Furthermore, it needs to be noted that the relatively short study period, coupled with the general stability of incomes, limits the chances of substantial income shifts being observed.

Contrary to our expectations (Hypothesis 3b), differences in life satisfaction associated with categorical inequalities generally persist after controlling for objective differences in wellbeing (Model 2). In fact the positive coefficients on subjective wellbeing for women, and even more so for Indigenous people, increase once objective wellbeing is accounted for. These findings imply life satisfaction adaptation for women and Indigenous respondents is partly masked when gender and Indigenous differences in objective wellbeing indicators are not accounted for. They also suggest differences in expectations and aspirations between gender and ethnic categories [62]. Finally, although moving to the petty bourgeoisie from other classes was associated with lower objective wellbeing on several measures, moving into the petty bourgeoisie from management, technical work or not working is associated with higher life satisfaction.

## Conclusions

### Objectives

This paper has provided one of the first longitudinal analyses of inequalities in objective and subjective wellbeing in Australia and one of the first internationally to link a broad set of objective wellbeing measures with subjective evaluations of life satisfaction. We have attempted to incorporate a more comprehensive set of measures of objective wellbeing than is typically studied in subjective wellbeing research, while also accounting for confounding factors such as positive and negative events in people’s lives. We also used multiple measures from the same individuals over time to decompose variation in objective and subjective wellbeing into between- and within-person components.

### Findings and implications

Our research shows a number of key findings. First, objective wellbeing is not equally distributed, even in a “highly developed” (on the HDI scale) and egalitarian society such as Australia. Women, Aboriginal people and Torres Strait Islanders, people from non-English speaking backgrounds and those in more disadvantaged occupational classes experience worse objective wellbeing than men, non-Indigenous Australians, English speaking Australians, and those in middle class jobs. There are also some more nuanced age differences in objective well- being. Gender, age, class, and ethnicity are sources of categorical inequality of the kind described by Tilly (1998) confirming our first hypotheses about structural sources of inequality.

Equally importantly, objective wellbeing is strongly associated with subjective life satisfaction. Better objective wellbeing implies higher life satisfaction and worse objective wellbeing implies lower life satisfaction. Thus even though there is a strong tendency for people in Australia and other Western societies to report high levels of life satisfaction [63], subjective wellbeing is strongly shaped by the objective conditions of people’s lives, as the subjective approach to wellbeing suggests [3].

The within-person effects show that life satisfaction varies as people’s economic and social circumstances change. We do not have enough data to know if short or long term changes in objective wellbeing have long term effects on subjective wellbeing (cf. [50]) but changes in objective wellbeing that are associated with differences in subjective wellbeing provide some evidence that individual life satisfaction is variable. This finding parallels cross-national comparative findings that population subjective wellbeing at the country level varies with country-level differences in objective wellbeing [64] and also indirectly supports arguments that temporal variations in life satisfaction imply that homeostatic set-points can be reset [50] and consequently people can undergo a more permanent shift in their default wellbeing position.

Furthermore, durable categorical inequalities, such as gender, Indigenous status and class have associations with life satisfaction that are independent of objective differences in wellbeing. Various mechanisms are likely at work here. Nussbaum [15] has argued that women’s generally high reported life satisfaction in many countries, despite objectively worse circumstances, is very likely due to adaptive preference formation—making do in the presence of a bad situation. A similar gender difference is typically found with respect to work satisfaction [65] and satisfaction with the gender division of labour in the home [66]. These findings are typically explained by adaptive preference formation and lower expectations among women than men, or by related arguments about women’s “intrinsic” or “constitutional” high levels of satisfaction [49]. Because we find large positive effects of Indigenous status on life satisfaction, when objective inequalities are controlled, a similar argument might apply to Australians from Aboriginal and Torres Strait Islander backgrounds many of whom experience the most profound disadvantage in Australia [31].

### Limitations and suggestions for further research

Although life satisfaction is grounded in the circumstances and experiences of people’s lives, our objective wellbeing measures do not capture all relevant elements of these circumstances. In addition to objective conditions, peoples’ lives are grounded in relationships of social evaluation, esteem and comparison in which they both judge and are judged. Our research does not consider how these social evaluation processes are related to life satisfaction. The persistence of categorical differences in life satisfaction after controlling for objective differences in wellbeing may reflect these kinds of unmeasured mechanisms, which if time varying, would not be controlled through our hybrid models. Our analyses could be extended by further research incorporating, for instance domain-specific indicators of life satisfaction, such as work satisfaction, relationship satisfaction, and economic satisfaction.

Another potential limitation of the study is that, in common with other social surveys, the objective circumstances are based on information provided by the respondent rather than through external sources (for instance, we use the income as reported by the respondents, rather than based on tax statements). This has the potential of introducing reporting bias into the analyses, whereby, for instance, more satisfied people also report better objective circumstances. While the fixed-effects part of our models provides a way of controlling for time-invariant factors, such as personality traits, which might be driving such reporting bias, the only way to fully eliminate the problem would be to rely on external objective data sources, such as government-collected administrative data, as the only source of information on the objective circumstances, a strategy that would be very difficult to implement in practice.

Our analyses are also limited by the short length of the panel. It is commonly accepted that individual life satisfaction is relatively stable unless affected by major life events such as repeated unemployment [51]or marriage [52]. Headey [50]has recently shown, using a 20 year panel, that a substantial proportion of individuals experience large long-term changes in life satisfaction. With more data we would be able to test this idea extensively, because we would observe more within-individual changes in objective wellbeing that could be linked to subjective wellbeing. Unfortunately we have only three waves of data for our research questions.

Finally, the very strong linkages between objective and subjective wellbeing imply that if we address objective differences in wellbeing we will also improve subjective wellbeing for many people. One of the critical target groups, for policy, however, is the segment of the population experiencing the most profound levels of objective disadvantage, that is, the lowest level of objective wellbeing on multiple indicators. In future research we intend to identify this group, based on their relative positioning on each of the objective measures, and track entry and exit into and out of this state. The policy responses to extremely low objective wellbeing are quite different if it is a temporary rather than enduring feature of people’s lives. The impacts of enduring low levels of objective wellbeing on life satisfaction and happiness are also likely to be profound, severely constraining the opportunities of the most disadvantaged members of society to live fulfilling and satisfying lives.
